# Adding a piece to the puzzle of Latin American blood donors and the potential risk of *Trypanosoma cruzi* transmission in Germany

**DOI:** 10.3389/fcimb.2022.1014134

**Published:** 2022-10-13

**Authors:** Julian Ullrich, Lutz Guertler, Ernst Quenzel, Franz Weinauer, Dieter Rößler, Ulrich Kalus, Axel Pruß, Pedro Albajar-Viñas, Michael Pritsch

**Affiliations:** ^1^ Division of Infectious Diseases and Tropical Medicine, University Hospital, LMU Munich, Munich, Germany; ^2^ Max von Pettenkofer-Institute for Hygiene and Medical Microbiology, LMU Munich, Munich, Germany; ^3^ BRK-Blutspendedienst, Munich, Germany; ^4^ Roche Diagnostics GmbH, Penzberg, Germany; ^5^ Institute of Transfusion Medicine, Charité University Hospital, Berlin, Germany; ^6^ Department of Control of Neglected Tropical Diseases, World Health Organization, Geneva, Switzerland; ^7^ German Center for Infection Research (DZIF), Partner Site Munich, Munich, Germany

**Keywords:** blood banks, blood donor screening, *Trypanosoma cruzi*, Chagas disease, transfusion, transmission, Germany

## Abstract

**Introduction:**

Chagas disease (CD) is caused by the *Trypanosoma cruzi* (*T. cruzi*) infection and has become a global health concern due to population mobility, as well as non-vectorial transmission routes. Several countries outside Latin America (LA) have reported transfusion-associated transmission, but equivalent studies in Germany are lacking. This study aims to collect first data on the risk of transfusion associated transmission as well as LA blood donors originating from CD endemic countries in Germany

**Materials and methods:**

A total of 305 blood donors who were assumed to be at risk for *T. cruzi* infection were retrospectively (267) as well as prospectively (38) selected at German blood donation sites in Bavaria and Berlin, and all retrospectively as well as 27 prospectively selected were serologically screened. Prospective study subjects additionally filled out a questionnaire.

**Results:**

All samples tested seronegative for *T. cuzi* specific antibodies. Prospectively enrolled study subjects all had high socio-economic status including good education. Knowledge regarding CD was limited but willingness to donate frequently was high. Blood donation rates from donors born in LA countries seem to increase from 2015.

**Discussion:**

Although no transfusion associated *T. cruzi* infection has been documented in Germany, it has likely already happened unnoticed, or will do in the near future. Performing risk-adapted serology-based blood donor screenings in Germany could avoid transfusion-associated transmission events as well as contribute to active case detection. Moreover, larger, and ongoing studies are needed to increase the evidence base as well as end the neglect of CD in Germany.

## Introduction

Chagas disease (CD) is a potentially fatal infection caused by *Trypanosoma cruzi* (*T. cruzi*). It is endemic in 21 Latin American (LA) countries with devastating effects on health and economies, possibly higher than that of other major infectious or chronic diseases (e.g. rotavirus or cervical cancer) (Lee et al., 2013). CD has become a global health issue due to population movements and several non-vectorial transmission routes, and is now prevalent in non-endemic areas such as Europe (Lidani et al., 2019). Moreover, it is one of the most neglected tropical diseases where less than 1% of those affected may receive adequate treatment even in rich countries such as Germany (Basile et al., 2011; Guggenbühl Noller et al., 2020).

Data concerning CD in European countries is scarce and patchy: Estimates state that roughly 4,6 million LA migrants live in Europe and that there may be between 68,000 to 122,000 undiagnosed cases of CD (Basile et al., 2011; Bayona-i-Carrasco and Avila-Tàpies, 2019). It is very difficult to identify the number of undocumented migrants although they may account for the highest CD prevalence rates (Jackson et al., 2010). Ongoing migratory waves, together with other population movements, such as travelers or adoptions, would require frequent and regular data assessment in order to provide a more accurate picture (Bayona-i-Carrasco and Avila-Tàpies, 2019). In 2020, a total of 140,565 immigrants from CD endemic LA countries were officially registered in Germany (Statista, 2021), undocumented migrants and migrants with European citizenship have to be counted on top of this.

Due to migrant groups participating in blood donation programs within their host countries, transfusion transmitted *T. cruzi* infections have already been described in non-endemic countries like Australia, Canada, Spain, and the USA (Angheben et al., 2015). Reacting to this, several countries (e.g. Belgium, France, Spain, Switzerland, and UK) have implemented nationwide screening of at-risk blood donors for *T. cruzi* specific antibodies (Angheben et al., 2015; Lidani et al., 2019). In Germany, only one study has been published so far with a total of 4,391 blood donors being screened as negative in 2015-2016 (Flores-Chavez et al., 2018). However, the study likely included only few to no blood donors at risk for CD. According to current regulations in Germany, blood donors including those at risk for CD are not screened serologically for *T. cruzi*. In 2009, recommendations by the German Advisory Committee Blood stated as reasons (i) the lack of data justifying such screening measures, (ii) the epidemiological situation and low prevalence of CD in Germany, and (iii) the low sensitivity as well as specificity of available screening tests (Arboprotozoea, 2009). Following regulations, individuals with a known *T. cruzi* infection and/or having received blood transfusions from CD endemic countries are excluded from blood donations. Additionally, individuals are put on hold for six months after visiting or living in malaria endemic regions, which excludes a relevant fraction of individuals at risk of acute CD due to the significant overlap of both protozoa (Arboprotozoea, 2009).

The primary aim of our study is to collect first data on the risk of transfusional *T. cruzi* transmission in Germany. Moreover, it should improve knowledge about the population of LA blood donors born in endemic countries for CD. For this, selected blood donors were retro- as well as prospectively screened for *T. cruzi* specific antibodies and personal information was prospectively collected on LA blood donors born in CD endemic countries *via* a questionnaire.

## Materials and methods

### Ethical considerations

The study protocol was approved by the Institutional Review Board at the Ludwig-Maximilians-University in Munich, Germany (opinion dated 19 September 2018, number 18-458) prior to study initiation and adhered to the most recent version of the Declaration of Helsinki.

### Retrospective study

We compiled a list of common Brazilian/Portuguese and/or Spanish surnames in LA by consulting multiple databases and national registries (**Supplemental Data 1**). We took spelling variants into account and excluded surnames also frequent in German (e.g. Jordan, Leon, Martin, or Reis). We utilized this in order to enrich the list of blood donors originating from LA countries with CD endemic geographic regions and thus those at increased risk of *T. cruzi* infection. For at least five years, EDTA plasma reserve samples of all active blood donors are kept in the Bavarian Red Cross central blood bank (Wiesentheid, Germany). Thus, in February 2019, we took plasma samples belonging to donors with listed names within the timeframe between January 2014 and January 2019 in a pseudonymized manner, including additional information on age range and sex.

### Prospective study

We recruited participants in the prospective study at different Bavarian Red Cross blood collection sites across Bavaria and the centralized blood transfusion services of the Institute of Transfusion Medicine, Charité University Hospital, Berlin between April 2019 and April 2020. We used the compulsory standard medical questionnaire prior to blood donations in order to identify donors aged 18 and older as well as born in one of the 21 LA countries with CD endemic geographic regions. We informed those potential study subjects about our study and enrolled all individuals that gave oral and written informed consent to the responsible medical doctor on site. Once enrolled, study subjects filled out a study questionnaire and we kept a 5 mL serum sample during the blood donation process which was stored at -20°C until screening for *T.cruzi* specific antibodies was performed.

### Screening for *T. cruzi* specific antibodies

We screened all retro- as well as prospectively collected plasma/serum samples for anti-*T. cruzi* IgG using the licensed Elecsys Chagas assay on a cobas e 411 analyzer by Roche diagnostics as described previously (Flores-Chavez et al., 2018). Sensitivity and specificity were shown to be 100.0% and 99.9%, respectively (Flores-Chavez et al., 2018), thus meeting the requirements of the Pan-American Health Organization guidelines concerning *T. cruzi* screening in hemotherapy (Organización Panamericana de la Salud, 2019). In addition to the standardized calibration of the cobas e analyzer prior to every test cycle, a total of five serum samples from known CD patients were added as positive controls in three distinct dilutions (5-, 10-, and 100-fold). A cut-off index for reactivity of 1.0 was used according to the instructions of the manufacturer.

### Data analysis

Pseudonymized data was entered in Microsoft Excel, anonymized once the necessity of look back procedures could be excluded, and then analyzed using SPSS version 27.

## Results

### Retrospective study

**Figure 1:**
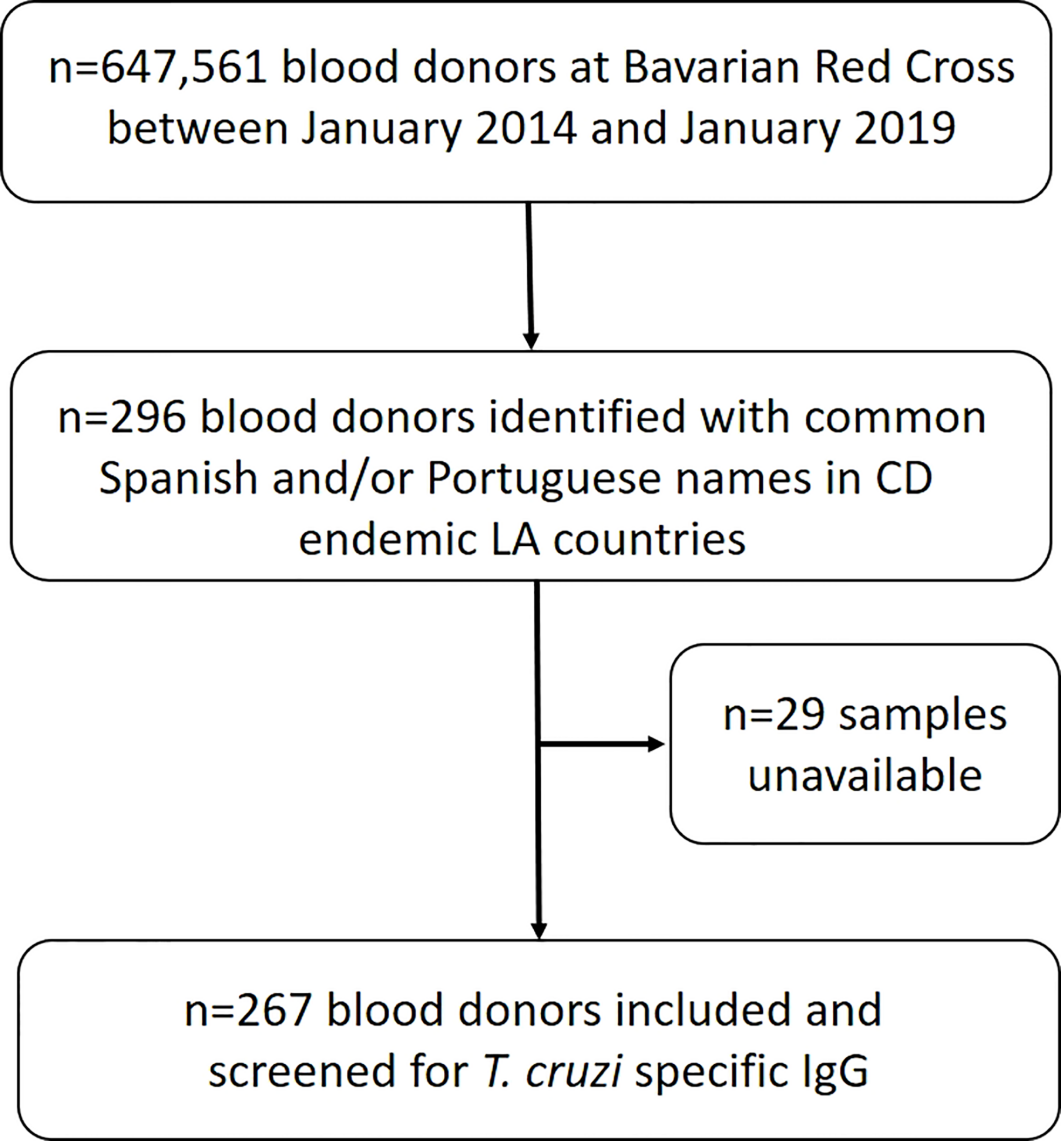
Selection algorithm of study subjects in the retrospective study.

From January 2014 to January 2019, a total of 3,514,501 blood donations were drawn from 647,561 donors by the Bavarian Red Cross at their donation sites. We selected a total of 296 donors (0.05%) by cross-checking the name list of common Brazilian/Portuguese and/or Spanish names in LA countries with CD endemic geographic regions (**Figure 1**; **Supplementary Data 1**). We were able to obtain 267 (90.2%) of the corresponding EDTA plasma reserve samples in order to perform *T. cruzi* antibody screening (**Figure 1**). Of those, 71/267 (26.6%) study subjects were selected for having frequent Brazilian/Portuguese names, 171/267 (64.0%) for frequent Spanish names, and 25/267 (9.4%) for names that are common in both languages. Sex was nearly equally distributed with 128/267 (47.9%) being females and 123/267 (46.1%) study subjects belonged to the age group 18-29 years at the time of blood donation. **Table 1** provides a more detailed description regarding age range and sex of the retrospective study population. All study subjects’ samples were non-reactive (highest measurement 0.26) for *T. cruzi* specific IgG, while all positive controls were reactive in all dilution steps.

**Table 1:** Age ranges and sex for retrospective study subjects.

	Female	Male	Total
**Age ranges**			
18-29	63 (51.2%)	60 (48.8%)	123
30-39	35 (48.6%)	37 (51.4%)	73*
40-49	26 (53.1%)	23 (46.9%)	49
50-59	11 (57.9%)	8 (42.1%)	19
60-69	3 (100.0%)	0 (0.0%)	3

*Sex information was unavailable for one study subject aged 30-39.

### Prospective study

**Figure 2:**
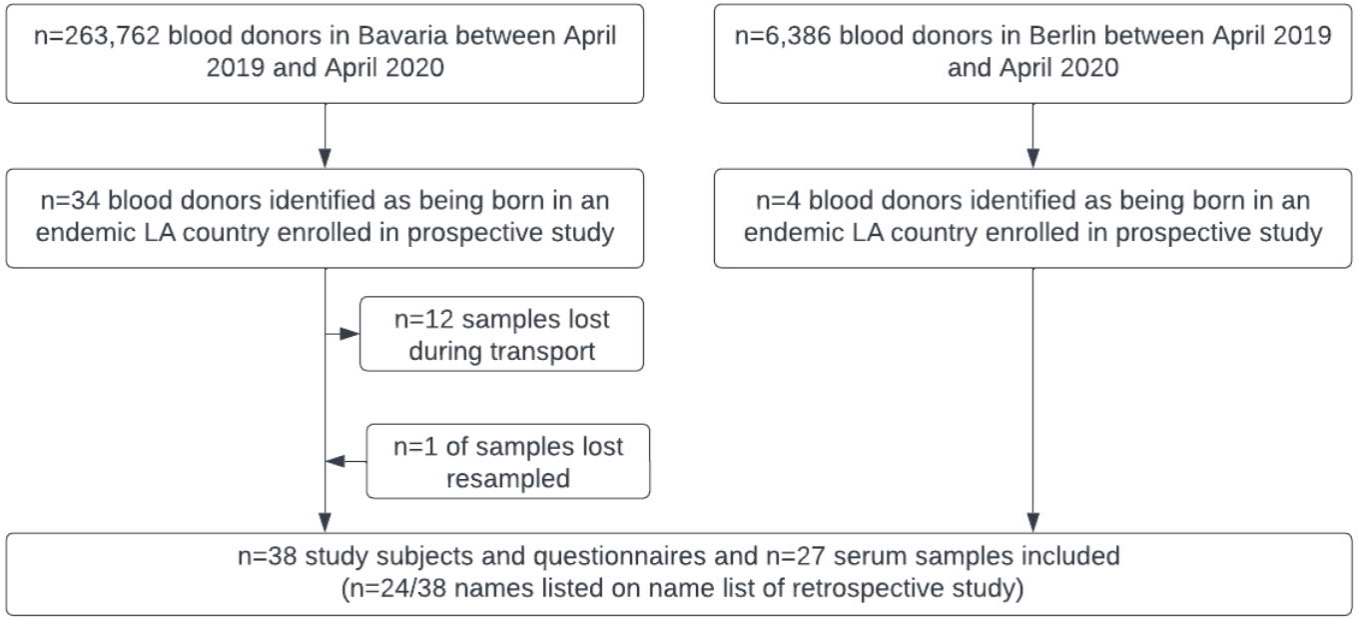
Selection algorithm of study subjects in the prospective study.

**Figure 3:**
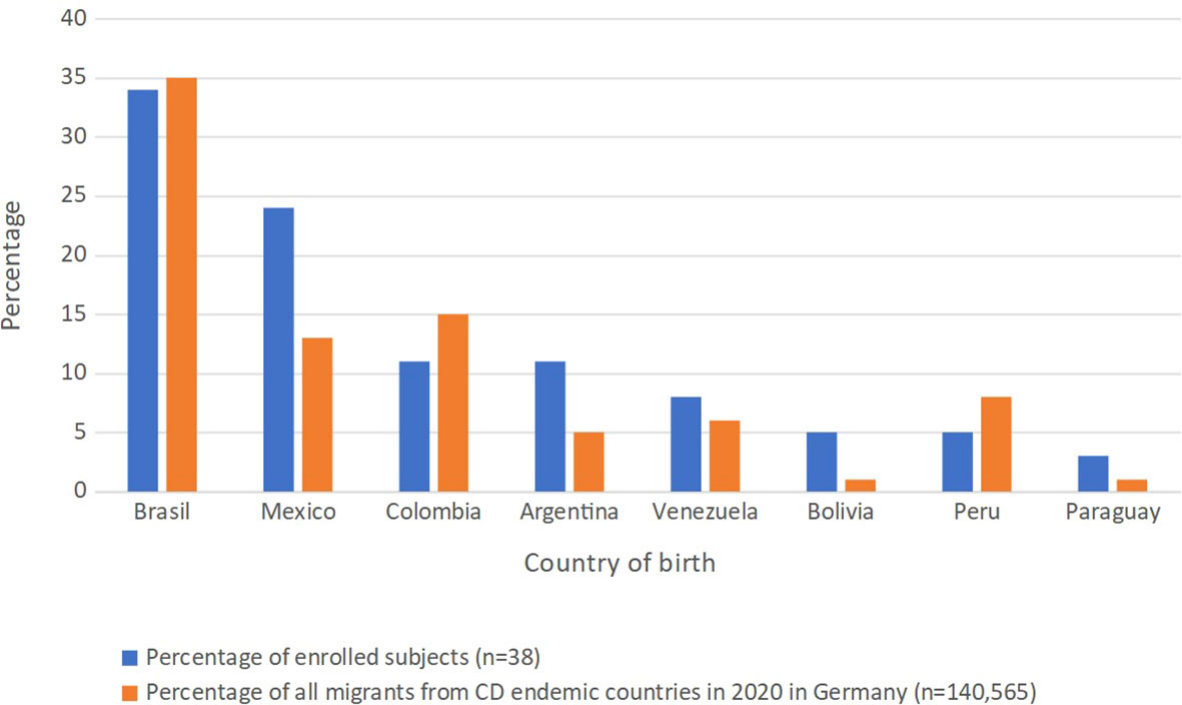
Comparison between the percentages of enrolled subjects and total registered immigrants from that country in Germany in 2020.

**Figure 4:**
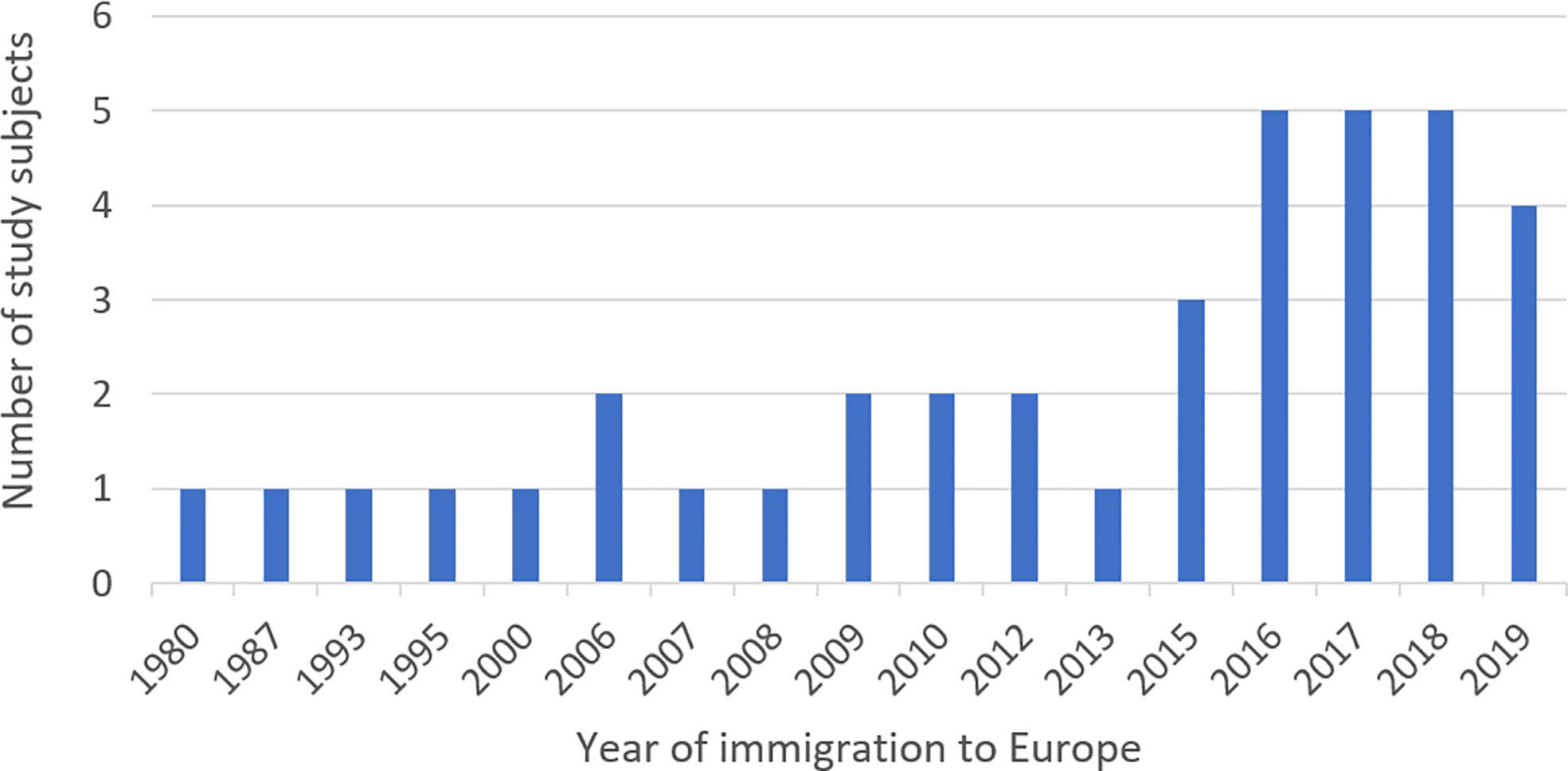
Year of immigration to Europe for all prospectively enrolled study subjects.

From April 2019 to April 2020, a total of 545,754 blood donations were drawn from 263,762 donors by the Bavarian Red Cross at all blood donation sites across Bavaria. In the same time frame, a total of 8,718 blood donations were drawn from 6,386 donors at the Institute of Transfusion Medicine, Charité University Hospital, in Berlin. A total of 38 study subjects could be enrolled in this prospective study consisting of 34 individuals from across Bavaria and 4 from Berlin (**Figure 2**). Of those, the names of 24/38 (63.2%) enrolled study subjects were included on the name list (**Supplementary Data 1**) used for the retrospective study and thus would have been detected by using this screening methodology. Questionnaire data was available for all 38 study subjects, while *T. cruzi* antibody screening could only be performed on 26 of them due to a transport associated loss of 12 serum samples. Donors of lost samples were contacted, asked to send a blood sample for testing, and one donor could be resampled. Of the 27 samples screened for *T. cruzi* specific IgG, all were non-reactive (highest measurement 0.11), while all positive controls were reactive in all dilution steps. **Table 2** provides information on study subjects in our prospective study. Most participants belonged to the age group 18-29 (22/38; 57.9%) and the study population consisted of slightly more males (23/37; 60.5%). Study subjects were born in a variety of countries with CD endemic geographic regions, with Brazil (13/38; 34.2%), Mexico (9/38; 23.7%), Argentina (4/38; 10.5%), and Colombia (4/38; 10.5%) being the most frequent (**Table 2**; **Figure 3**). Two study subjects were born in Bolivia, the country with the highest prevalence of CD per 100,000 inhabitants worldwide, and both tested serologically negative. As expected, considering the country of origin, more study subjects stated Spanish as their mother tongue (24/38; 63.2%) than Portuguese (14/38; 36.8%). The vast majority of study subjects (37/38; 97.4%) grew up in a LA country with CD endemic geographic regions and nearly all of them resided there (36/37; 97.3%) prior to emigrating to Germany. Most study subjects kept their nationality of origin (32/38; 84.2%), whereas some (6/38; 15.8%) adopted the German nationality in addition to their original nationality. Most study subjects (22/38; 57.9%) immigrated to Germany from 2015 to 2019, the others immigrated between 1980 and 2013 (**Figure 4**). All donors grew up in larger cities with more than 100,000 inhabitants and were living in houses made out of stone or concrete. A total of 24/37 (64.9%) had heard about CD in their country of birth, but only 8/38 (21.1%) could correctly describe three typical symptoms. Some 21/30 (70.0%) correctly indicated that CD could possibly be transmitted *via* the triatomine feces but only 12/30 (40.0%) knew that it could be transmitted *via* transfusion of blood and blood derivates, while 6/38 (15.8%) did not know any ways of transmission. Only 9/38 (23.7%) had seen a triatomine bug during their housing in LA and 2/37 (5.4%) knew someone suffering from CD in their LA country of birth. Most study subjects (25/38; 65.8%) had already donated blood prior to study inclusion and of those, 7/25 (28.0%) had donated in Germany and 18/25 (72.0%) in LA, respectively. All study subjects were highly educated and socio-economically advantaged: All had finished at least secondary schooling and a total of 24/38 (63.2%) were in possession of a university degree.

**Table 2:** Questionnaire data of prospective study subjects.

	Female	Male	Total
**Age range**			
18-29	7	14	22*
30-39	2	8	10
40-49	2	1	3
50-59	1	0	1
60-69	2	0	2
**Country of birth**			
Brazil	6	7	13
Mexico	1	7	9*
Colombia	0	4	4
Argentina	1	3	4
Venezuela	2	1	3
Bolivia	2	0	2
Peru	2	0	2
Paraguay	0	1	1
**Arrival in Europe**			
2019-2020	1	3	4
2017-2018	4	5	10*
2015-2016	3	5	8
2010-2013	1	4	5
2000-2009	1	6	7
1980-1999	4	0	4
**Highest educational status**			
Secondary school	5	5	10
Vocational training	2	2	4
University degree	7	16	24*
**Has seen a triatomine bug inside their housing in LA**			
Yes	2	6	9*
No	12	17	29
Knows someone with CD in LA**			
Yes	1	1	2
No	13	21	35*
Heard about CD in country of birth**			
Yes	10	14	24
No	4	8	13*
**Ways of CD transmission**			
Triatomine bug	6	15	21
Sexual intercourse	1	2	3
Blood transfusion	5	7	12
Mosquito	1	3	4
Mother to child	1	3	4
Juice consumption	1	1	2
Organ transplantation	1	5	6
Physical contact	1	2	3
Don’t know	1	4	6*
No answer	4	4	8
Infected can feel healthy**			
Yes	6	6	12
No	1	3	4
Don’t know	7	13	20
**Most common CD symptoms**			
No correct symptom	9	14	24*
1 correct symptom	1	1	2
2 correct symptoms	0	4	4
3 correct symptoms	4	4	8
**Would donate organs**			
Yes	13	19	33*
No	0	0	0
Don’t know	1	4	5
**Previously donated blood**			
In country of origin	8	9	18*
In Germany	1	6	7
No	5	8	13
**Received blood transfusion(s)**			
Yes	1	0	1
No	13	23	37*

*Sex was unavailable for one study subject aged 18-29.

**Not all participants answered those questions so numbers don’t add up to 38.

CD, Chagas disease; LA, Latin American.

## Discussion

To the best of our knowledge, this study is the first to examine blood donors from LA countries with CD endemic geographic regions and the associated risk for *T. cruzi* transmission in Germany. No *T. cruzi* infections were detected among the 293 screened blood donors. Several European countries such as Spain, the UK, Switzerland and France have implemented screenings of blood donors at risk for *T. cruzi* infection (Angheben et al., 2015). Studies carried out in Spain and France have shown a prevalence of at risk blood donors to be 0,66% in Spain and 0,31% in France, respectively (Piron et al., 2008; El Ghouzzi et al., 2010). In addition, several transfusion-associated *T. cruzi* infections have been described in non-endemic countries (including several European countries), of which some were detected after the implementation of serological blood donor screenings and associated look-back procedures (Flores-Chávez et al., 2008; Kessler et al., 2013; Angheben et al., 2015; Blumental et al., 2015; Ries et al., 2016).

As we had no possibility to identify the risk for CD or the country of birth in retrospect, we used a list of common Brazilian/Portuguese and/or Spanish names in CD endemic LA countries as a proxy for LA origin that resulted in screening 267 blood donors who had donated over a time period of five years. By applying the same name list to our 38 prospective study subjects, a total of 24 (63.2%) would have been detected retrospectively. We therefore assume that the 267 retrospectively identified blood donors include a significant fraction of individuals who were actually born in CD endemic LA countries. A limitation of this approach is that LA blood donors with high risk of *T. cruzi* infection could have been missed due to the exclusion of surnames also common in Germany or name changes (e.g. after marriage or adoption) and Portuguese as well as Spanish citizens without risk could have been falsely included.

In the prospective study, all 38 study subjects who enrolled over a time period of 12 months were born in a CD endemic LA country. We could not identify the number of blood donors born in a LA country with CD endemic geographic regions who were identified but refused to take part in our prospective study. As this was not included in the original study protocol and we were unable to gather this information in retrospect, it has to be mentioned as a shortcoming. The small number of blood donors and blood donation centers included and some samples being lost during transport also impacts the informative value of our study.

One of the strengths of our prospective study is that we collected additional data on study subjects *via* questionnaires. Higher socio-economic status is correlated with less risk for *T. cruzi* infections (Hotez and Gurwith, 2011): With 24 out of the 38 prospectively enrolled study subjects (63.2%) having a university degree and the rest having at least finished secondary schooling, study subjects all had high socio-economic status. In addition, all 38 study subjects grew up in cities >100,000 inhabitants situated in housing with little risk of insect vector colonization and only two reported having known someone suffering from CD in their country of birth. The country of birth also influences the likelihood of CD infection (Bern et al., 2019) and only two of our participants were born in Bolivia. In the light of this information, it is not surprising that no infection was detected in our prospective cohort. Enrolled subjects from Mexico, Argentina, Bolivia and Paraguay were slightly overrepresented percentagewise in our study cohort (**Figure 3**). Subjects from Colombia and Peru were slightly underrepresented and the biggest subject group from Brazil was accurately represented (**Figure 3**). However, the sample size was too small in order to draw relevant conclusions out of these differences.

A way of reducing the already limited likelihood of transfusional as well as transplantational *T. cruzi* transmission in Germany is to raise awareness about CD among individuals at risk for CD, e.g. LA migrants or travellers having lived in LA countries at risk of CD that are or eventually could become donors. Once diagnosed with CD, they would be excluded from donations and thus render this method of transmission unlikely. Among our prospectively enrolled study subjects ─having both high socio-economic status and education─ general knowledge about CD was low: e.g. most study subjects (24/38; 63.2%) could not even correctly mention one common symptom of CD and 13/37 (35.1%) study subjects hadn’t heard of CD in their country of birth. These findings are in agreement with a previous study performed among Bolivians living in Munich, Bavaria, Germany (Navarro et al., 2017). Continuing efforts to raise awareness about CD with at-risk individuals as well as health care personnel might lead to increased diagnoses and with this to a decreased risk of infection transmission. It is likely that less than 1% of individuals at risk of CD receive adequate care in Germany at the present time (Basile et al., 2011; Guggenbühl Noller et al., 2020). All of this data forms a strong argument for targeted, innovative, as well as continued information, education, and communication campaigns in regard to CD.

The at-times rapidly changing landscape of migratory movements poses challenges to blood banks and other institutions alike. The number of donors should be maximized while maintaining the highest levels of hemo-vigilance. Although there has never been a documented case of transfusional *T. cruzi* transmission in Germany, we should take heed of the aphorism “absence of evidence is not evidence of absence”: That it cannot be ruled out that transfusional transmission has likely taken place or that it could take place in the future. That no case had been documented to date might easily be due to the absence of appropriate donor screening, as seen in other European countries (Angheben et al., 2015). The increasing popularity of Germany among LA migrants and their participation in the blood donation system (Navarro et al., 2017), makes a strong case for the undertaking of additional and larger investigations like this one, as well as for direct implementation of risk-adapted serology-based blood donor screening in Germany. These measures could avoid potentially fatal transfusion-associated CD as well as contribute to active case detection and thus help to end the neglect of CD in Germany. Germany, together with all other member states of WHO, endorsed the new road map for neglected tropical diseases 2021-2030 in the 73rd World Health Assembly in November 2020 (WHO, 2020). One of the objectives is the verification of the interruption of transfusional *T. cruzi* transmission and by implementing an appropriate national protocol Germany could verify this much before 2030.

In summary, this first attempt to describe the German landscape of LA blood donors born in countries with CD endemic geographic regions suggests a generally higher socio-economic status and thus reduced overall risk of *T. cruzi* infection compared to the general population in their countries of birth. Although, no transfusion associated *T. cruzi* infection has been documented in Germany so far, it likely could have already taken place unnoticed or will do in the near future. A risk-adapted serology-based blood donor screening in Germany could avoid transfusion-associated *T. cruzi* transmission, maximize the number of donors, as well as contribute to active case detection and thus help to end the neglect of CD in Germany. At the very least, more and larger studies are needed to increase the evidence base. The rapidly changing landscape of migratory movements remains challenging and calls for constant surveillance until CD is eliminated or no longer a neglected tropical disease.

## Data availability statement

The original contributions presented in the study are included in the article/**Supplementary Material**. Further inquiries can be directed to the corresponding author.

## Ethics statement

The studies involving human participants were reviewed and approved by institutional review board at the Ludwig-Maximilians-University in Munich, Germany. The patients/participants provided their written informed consent to participate in this study.

## Author contributions

LG and MP provided funding for the study. LG and MP designed the study. EQ, FW, UK, AP, and PA-V commented on the study protocol. JU was responsible for recruitment with the help of LG, EQ, FW, UK, AP, and MP. DR supervised laboratory analyses. JU analyzed the data with the help of LG and MP. JU drafted the first manuscript version. All authors contributed to the article and approved the submitted version.

## Acknowledgments

We thank all study participants for participation and Roche Diagnostics GmbH for providing serological analyses free of charge.

## Conflict of interest

Author DR is employed by Roche Diagnostics GmbH, Penzberg, Germany.

The remaining authors declare that the research was conducted in the absence of any commercial or financial relationships that could be construed as a potential conflict of interest.

## Publisher’s note

All claims expressed in this article are solely those of the authors and do not necessarily represent those of their affiliated organizations, or those of the publisher, the editors and the reviewers. Any product that may be evaluated in this article, or claim that may be made by its manufacturer, is not guaranteed or endorsed by the publisher.

## Supplementary material

Supplementary Data Sheet 1 List of common Brazilian/Portuguese and/or Spanish surnames in CD endemic Latin American countries.
