# Supplementary material for: Adding a piece to the puzzle of Latin American blood donors and the potential risk of Trypanosoma cruzi transmission in Germany
Source: Front Cell Infect Microbiol. 2022 Oct 13;12:1014134. doi: 10.3389/fcimb.2022.1014134 (PMC9606580; doi:10.3389/fcimb.2022.1014134)
Supplement: Supplementary Data Sheet 1 — List of common Brazilian/Portuguese and/or Spanish surnames in CD endemic Latin American countries. [file DataSheet_1.docx]

**Supplementary data 1**

In order to retrospectively select blood donors potentially born in or to mothers from CD endemic LA countries with increased risk for *T. cruzi* infection, we compiled a list of common Brazilian/Portuguese and/or Spanish surnames in CD endemic LA countries.

As Brazil is the only Portuguese speaking country in LA which is endemic for CD, we included 100 frequent Brazilian surnames. The surname Reis was also frequent among German names and thus removed from the list.

For Spanish names, we initially included 226 frequent surnames in CD endemic LA countries. However, we subsequently removed the names Jordan, Leon, Martin, and Martins due to overlap with German names.

A total of 22 surnames overlapped between the lists of Spanish (222) and Brazilian/Portuguese (99) surnames. Thus, the final list of very common Brazilian/Portuguese and/or Spanish surnames in CD endemic LA countries possessed 299 surnames.

Common Brazilian/Portuguese surnames in Brazil (n=77):

Abreu, Almeida, Alves, Amaral, Amorim, Anjos, Antunes, Araujo, Assuncao, Azevedo, Baptista, Barbosa, Barros, Batista, Borges, Branco, Brito, Cardoso, Carneiro, Carvalho, Coelho, Correia, Costa, Crunha, Esteves, Faria, Ferreira, Figueiredo, Fonseca, Freitas, Gaspar, Goncalves, Guerreiro, Henriques, Jesus, Leal, Leite, Loureiro, Lourenco, Macedo, Machado, Magalhaes, Maia, Matias, Matos, Meideros, Melo, Monteiro, Moraes, Moreira, Mota, Moura, Nascimento, Neto, Neves, Nogueira, Oliveira, Pacheco, Paiva, Punheiro, Pinho, Pires, Ramos, Raposo, Ribeiro, Santana, Silva, Simoes, Soares, Souza, Sa, Tavares, Teixeira, Valente, Vaz, Vicente, Vieira

Common Spanish surnames in CD endemic LA countries (n=200):

Acosta, Aguilar, Aguilera, Aguirre, Alarcon, Aliaga, Alonso/Alonzo, Alvarez, Anez, Antelo, Antezana, Apaza, Aramayo, Arancibia, Aranibar, Araya, Arce, Arias, Arteaga, Avila, Ayala, Baez, Balderrama, Barrientos, Bejarano, Beltran, Benitez, Blanco, Bustillos, Caballero, Cabrera, Caceres, Calderon, Calle, Camacho, Cardenas, Cardozo, Carlos, Carrasco, Carvajal, Castillo, Cespedes, Chambi, Chavez, Choque, Claros, Claure, Coca, Colque, Condori, Contreras, Cordova, Cortes/Cortez, Cossio, Crespo, Cuellar, Daza, Delgadillo, Delgado, Dorado, Duran, Encinas, Escobar, Espinosa/Espinoza, Ferrufino, Flores, Franco, Fuentes, Galeano, Gaviria, Gil, Gimenez, Gonzales, Gutierrez, Guzman, Herbas, Heredia, Hernandez, Herrera, Hinojosa, Huanca, Hurtado, Ibanes/Ibanez, Iglesias, James, Jimenes/Jiminez, Justiniano, Landivar, Laura, Ledezma, Limachi, Llanos, Loayza, Loza, Luna, Maldonado, Mamani, Martinez, Medina, Medrano, Mejia, Melgar, Menacho, Mendoza, Mercado, Molina, Montano, Montero, Morales, Morell, Moreno, Moscoso, Munoz, Murillo, Navarro, Nina, Nogales, Orellana, Ortega, Ortis/Ortiz, Pacheco, Padilla, Paniagua, Parada, Paredes, Paz, Pena, Peredo, Perez, Poma, Ponce, Ponze, Prado, Quiroga, Quiros/Quiroz, Quisbert, Quispe, Ramirez, Ramos, Reyes, Ribera, Rios, Rivera, Rivero, Roca, Rojas, Romero, Rubio, Ruiz, Salas, Salazar, Salinas, Salvatierra, Sanches/Sanchez, Sandoval, Sanz, Saravia, Saucedo, Sejas, Sepulveda, Serrano, Siles, Silva, Soliz, Soria, Sosa, Soto, Suares/Suarez, Tapia, Teran, Terceros, Terrazas, Ticona, Ticonas, Torrico, Vaca, Valdes/Valdez, Valdivia, Valencia, Valenzuela, Valverde, Vargas, Vasquez, Vega, Veizaga, Velarde, Velasco, Velasques/Velazquez, Vera, Villalba, Villarroel, Villca, Villegas, Zabala, Zambrana, Zapata, Zeballos, Zembrano, Zenteno, Zurita

Common Brazilian/Portugues and Spanish surnames used in CD endemic LA countries (n=22):

Andrade, Campos, Castro, Cruz, Dias/Diaz, Domingues/Dominguez, Duarte, Fernandes/Fernandez, Garcia, Gomes/Gomez, Lima, Lopes/Lopez, Marques/Marquez, Mendes/Mendez, Miranda, Nunes/Nunez, Pereira, Pinto, Rocha, Rodrigues/Rodriguez, Santos, Torres
